# The search for invertebrate consciousness

**DOI:** 10.1111/nous.12351

**Published:** 2020-08-30

**Authors:** Jonathan Birch

**Affiliations:** The London School of Economics and Political Science

## Abstract

There is no agreement on whether any invertebrates are conscious and no agreement on a methodology that could settle the issue. How can the debate move forward? I distinguish three broad types of approach: theory-heavy, theory-neutral and theory-light. Theory-heavy and theory-neutral approaches face serious problems, motivating a middle path: the theory-light approach. At the core of the theory-light approach is a minimal commitment about the relation between phenomenal consciousness and cognition that is compatible with many specific theories of consciousness: the hypothesis that phenomenally conscious perception of a stimulus facilitates, relative to unconscious perception, a cluster of cognitive abilities in relation to that stimulus. This “facilitation hypothesis” can productively guide inquiry into invertebrate consciousness. What is needed? At this stage, not more theory, and not more undirected data gathering. What is needed is a systematic search for consciousness-linked cognitive abilities, their relationships to each other, and their sensitivity to masking.

## 1 The Bats and the Bees

In Nagel’s (1974) “What is it like to be a bat?”, the focal example is well chosen. A bat navigates the world using a sense foreign to most of us, echolocation.^1^ This creates an immediate sense of distance: most of us can’t imagine what it’s like to do that. Yet bats, being mammals, are evolutionarily close enough to humans for us to readily accept that there is *something* it’s like to be a bat. It’s just that we struggle to imagine what it is. With bees, it’s rather the opposite. Bees navigate the world using broadly familiar sensory apparatus: vision and olfaction. Yet because they are so evolutionarily distant from humans, and because their nervous system is so radically differently organized and contains so many fewer neurons (approximately 1 million, compared with our approximately 100 billion), there has been serious debate about whether there is *anything* it’s like to be a bee.

There are those who defend the idea of insect consciousness^2^, including Barron and Klein (Barron & Klein, 2016; Klein & Barron, 2016a), Tye (2016a, 2016b), Feinberg and Mallatt (2016, 2018, 2020) and Ginsburg and Jablonka (2019). There is also a vocal opposition including Adamo (2016a, 2016b), Allen-Hermanson (2008, 2016), Key, Arlinghaus, and Browman (2016), and Hill (2016). They argue that the evidence points to insects being “natural zombies”: cognitively sophisticated creatures with no conscious experiences. The argument often (though not always) takes the form: yes, they do many cognitively impressive things, but we could also design a robot that could do those things, and we wouldn’t think the robot was thereby conscious.

There is no agreement about whether insects are conscious or not, and, more fundamentally, no agreement on a methodology that could settle the issue. The same problem arises in other disputed cases of consciousness in invertebrates: cephalopod molluscs (Godfrey-Smith, 2016; Mather, 2007, 2019) and decapod crustaceans (Birch, 2017; Elwood, 2012). Though my focus in this article is on evidence from invertebrates, debates of a very similar character occur in relation to fish (see Key, 2016 and the 46 associated commentaries). There is a deep methodological problem here, and it arises in all cases where there is serious debate as to whether an animal has any conscious experiences at all. This is the problem I aim to address.

This article will revolve around a distinction between three types of methodological strategy: *theory-heavy, theory-neutral* and *theory-light.* The theory-heavy approach is this: We start with humans. We develop a well-confirmed, complete theory of consciousness in humans, and we take this theory “off the shelf” and apply it to settle the question of whether animals, in disputed cases, are conscious or not. This approach was advocated by Dennett in the 1990s:

How, though, could we ever explore these “maybes”? We could do so in a constructive, anchored way by first devising a theory that concentrated exclusively on human consciousness—the one variety about which we will brook no “maybes” or “probablys”—and then look and see which features of that account apply to which animals, and why. (Dennett, 1995, p. 700)

We can contrast this with the theory-neutral approach, which starts with the assumption that the theory-heavy approach will not work. We should *not* start with a theory of human consciousness: these theories are all too speculative and controversial, and we are too far away from achieving consensus. What we should do instead is build up a list of the behavioural, functional and anatomical similarities between humans and non-human animals, and use arguments from analogy and inferences to the best explanation to settle disputes about consciousness. Once we have answers regarding the distribution of consciousness in nature, we might then be in a position to build a better theory.^3^

I will argue that the theory-heavy and theory-neutral approaches are both unpromising, motivating the need for a middle path between them. The middle path I call the *theory-light* approach. The theory-light approach aims to capture what the theory-heavy and theory-neutral approaches get right, while avoiding their pitfalls.

## 2 The Theory-Heavy Approach: Global Workspace Theory

Let’s start by considering the theory-heavy approach. There are many theories one might try to take off the shelf and apply to questions of animal consciousness. Here I will focus on two examples: the global workspace theory of Baars, Dehaene and collaborators (Baars, 1989, 2017; Dehaene, 2014; Dehaene & Changeux, 2011), and Merker’s (2005, 2007) midbrain theory. I have chosen these examples not just because they have been prominent in the recent debate, but also because, taken together, they help us see a wider dilemma faced by theory-heavy approaches.

The global workspace theory posits the existence in the brain of a global broadcast mechanism that integrates representations from perceptual systems, affective systems and memory systems and broadcasts the integrated content back to the input systems and onwards to a wide variety of consumer systems, including mechanisms of verbal report, planning, reasoning and decisionmaking (Dehaene & Changeux, 2011, p. 209). The representations currently being broadcast are said to be in the global workspace. In humans, the broadcast mechanism is widely thought to be dependent on the cortex, and specifically on the prefrontal cortex (Dehaene & Changeux, 2011). Interpreted as a theory of phenomenal consciousness and not just cognitive access, the theory states that a representation becomes phenomenally conscious when it enters the workspace, whereas more localised processing outside the workspace occurs without phenomenal consciousness.

The empirical case for the existence of such a mechanism rests on well-established experimental paradigms (especially backward masking and the attentional blink) that allow us to compare, using neuroimaging, the processing that results from unconscious perception of a stimulus with the processing that results from conscious perception of the same stimulus (Dehaene, 2014). These studies rely on the assumption that healthy adult humans can accurately report their experiences, allowing the masking or attentional blink protocol to be calibrated against verbal reports.

Philosophers tend to worry not so much about the existence of the mechanism as about its hypothesized constitutive link to phenomenal consciousness. Could there not be phenomenally conscious states outside the workspace? What methodological approach could settle this question, given that states outside the workspace would not be available to voluntary report? This is the territory of the long-running “overflow” debate (see Overgaard, 2018; Phillips, 2018 for critical reviews of recent work). Let’s suppose, for now, that the debate has been resolved in the case of adult humans who can report their experiences. Suppose that, with respect to such humans, the global workspace theory is an extensionally adequate theory of phenomenal consciousness, in that all and only those states that enter the global workspace are phenomenally conscious. It would not thereby resolve questions about disorders of consciousness and about non-human animals, for reasons I will now explain.

What does global workspace theory say about these cases? The theory is most naturally read as saying nothing. The theory says: in adult humans with a capacity to report their experiences, entry to the global workspace confers phenomenal consciousness on mental representations. It is a theory of how the conscious/non-conscious distinction is drawn in healthy individuals of one species. Interpreted this way, the theory is silent on the question of the sufficient conditions for consciousness in humans with disorders of consciousness or in non-human animals. Let’s call this the “cautious interpretation” of the theory.

If the theory is interpreted in this way, it clearly cannot serve as the basis for a theory- heavy approach to animal consciousness (or, for that matter, to infant consciousness or to the minimally conscious state). A theory-heavy approach requires us to interpret the theory in a less cautious way. One option here is to interpret the theory as claiming that, *in general,* entry to a global workspace confers phenomenal consciousness on mental representations, and that the presence of a global broadcast mechanism, in whatever context it is found, provides positive evidence of the presence of phenomenal consciousness. Let’s call this the “ambitious interpretation” of the theory.^4^ A recent article by Dehaene, Lau, and Kouider (2017) on AI consciousness hints that Dehaene may be sympathetic to the ambitious interpretation.

In taking this route, however, we run into a serious interpretative difficulty, as Carruthers (2018a, 2018b) has observed. Outside of the paradigm case of a healthy adult human, what qualifies as a *global* broadcast mechanism of the kind that confers phenomenal consciousness? “Global broadcast” implies a wide range of consumer systems. If a single consumer system (say, verbal report or theory of mind) is taken offline, do we still have a global broadcast network that suffices to confer phenomenal consciousness on its entrants? What if two consumer systems (say, both verbal report and theory-of-mind) are taken offline? How much of the network of receivers can be knocked out and still leave behind a network that is sufficient for generating phenomenal consciousness? We don’t want to say that *any* form of broadcast network will do, regardless of the input sources and the consumer systems, because then we will end up concluding that very simple robots are conscious. But we also have no reason to insist that the full set of human consumer systems, including forms of linguistic, normative and social cognition, is required.

What we find is that, on this second-order question, even the ambitious formulation of global workspace theory is silent. On the face of it, there is no principled way of augmenting the theory with an account of which parts of the human global broadcast network are dispensable for phenomenal consciousness and which are indispensable. To construct such an account, we would first need a clear picture of which non-human animals and brain-damaged humans are conscious and which are not, and this is precisely the issue we were hoping the theory could settle.^5,6^

In sum: interpreted cautiously, global workspace theory tells us that the global broadcast network of a healthy adult human is sufficient for consciousness, but remains silent about cases in which something less than the full network is present. Interpreted ambitiously, it tells us that all non-human animals with whom we share all the indispensable features of the human global broadcast network are conscious, but its human evidence base leaves it with no principled way of answering the question of which features are indispensable.

## 3 The Theory-Heavy Approach: The Midbrain Theory

Let’s turn to a different version of the theory-heavy approach. Barron and Klein’s (2016) argument for insect consciousness starts with Merker’s (2005, 2007) theory, which locates the basis of human consciousness not in the cortex, as recent formulations of the global workspace theory do^7^, but in the midbrain, a much more evolutionarily ancient, sub-cortical part of the brain.

Merker’s argument is that the integrative mechanisms of the midbrain are sufficient for conscious experience even in the absence of a cortex. A particularly important role is given to a part of the midbrain called the superior colliculus. Barron and Klein apply Merker’s theory to insects: they argue that there are mechanisms in the insect closely analogous to the superior colliculus, performing the same functions related to the integration of sensory inputs and orientation to external objects. They point in particular to a region called the central complex that lies between the mushroom bodies in the insect brain. They argue: both the superior colliculus and the central complex have the function of modelling the moving animal in its environment, drawing on multiple sources of sensory input and outputting to motor systems. In vertebrates, these midbrain mechanisms suffice for consciousness, so we should infer that the analogous mechanisms in the insect brain also suffice. This is an argument from analogy, of a kind, but it is backed by a specific theory of what conscious experience is and what it does.

The difficulty for this approach is that the human evidence base for Merker’s theory is weak, especially in contrast with the evidence base for the global workspace theory. To support a theory that says the superior colliculus suffices for consciousness even in the absence of a cortex, one must find cases in which reliable evidence of consciousness can be obtained from subjects with no functional cortex. Merker (2007) relies heavily on cases of hydranencephaly: tragic cases of children who are born with severe cortical damage. Merker interviewed the carers of many such people and analysed thousands of the carers’ emails, and argued on the basis of this evidence that children with hydranencephaly display reactions that suggest conscious experiences. The children oriented towards stimuli; they seemed to recognize objects and people, and they had apparent emotional responses to people they recognized, such as their siblings. Note here the role played by a free-standing argument from analogy: these children displayed outward behaviours that, in healthy children, would normally have a conscious experience among their causes (although even this assumption might be disputed). This argument from analogy is *not* backed by an independently well-supported theory: it is supposed to form part of the evidential base for such a theory.

This type of case faces two serious problems. One is that patients with hydranencephaly have varying degrees of remaining cortical tissue. Critics have pressed on this, arguing that, to the extent that these behaviours are evidence of consciousness at all, they are evidence of processes occurring in the remaining cortical tissue (Watkins & Rees, 2007). The other is that Merker must explain away widely accepted evidence of blindsight (Weiskrantz, 1986), whereby people with damage to the primary visual cortex subjectively report having no conscious experience in a particular region of the visual field, yet still perform better than chance in forced choice tasks that require access to information in that region. The midbrain is intact in these patients, and the cortex is damaged. If the patients’ verbal reports are believed, conscious visual experience is dependent on the primary visual cortex, and a functional midbrain does not suffice for it (an objection posed to Merker by Doesburg & Ward, 2007; Piccinini, 2007; Schlag, 2007; Watkins & Rees, 2007).

Barron and Klein (2016) also cite a different line of evidence in support of Merker’s theory: neuroimaging studies of patients emerging from anaesthesia (in particular, Långsjö et al., 2012) which show an important role for midbrain structures. However, this type of evidence faces problems that parallel those discussed above. First, while Långsjo and colleagues’ results highlight the importance of the midbrain in restoring consciousness, they also highlight the importance of the anterior cingulate cortex (ACC) and connections between the ACC and parietal and frontal cortical regions (a criticism raised by Allen-Hermanson, 2016). They describe this as “only minimal cortical activity”, and Klein and Barron (2016b, pp. 5-6) stress how minimal it is, but (as in cases of hydranencephaly) it precludes any clear-cut demonstration of consciousness without cortical activity. Second, a subject in the process of waking from anaesthesia, but not yet fully awake, cannot report their experiences. When subjects opened their eyes in response to a command, Långsjö et al. (2012, p. 4936) took this as evidence of consciousness. It is evidence of responsiveness, but what is at stake here is whether these subjects were having phenomenally conscious experiences—experiences it felt like something to undergo—at the time the measurements were made. Since unconscious subjects sometimes respond to simple verbal commands (Kouider, Andrillon, Barbosa, Goupil, & Bekinschtein, 2014), a subject opening their eyes on command is not strong evidence of conscious experience. Here too, a free-standing argument from analogy is needed to bridge the gap: a fully awake, conscious human who is told to open their eyes will consciously hear the command and respond, so we are to infer that a human emerging from anaesthesia who responds to the same command has also consciously heard it.

In sum, one has no reason to be convinced by Barron and Klein’s argument unless one also has reason to believe Merker’s theory, and to be convinced by the evidence for Merker’s theory one must be willing to accept free-standing arguments from analogy from healthy to brain-damaged humans, or from fully awake humans to humans emerging from anaesthesia. Those sceptical of this kind of evidence for the midbrain theory as a theory of human consciousness will, inevitably, be unmoved by its application to insects.

Our two examples help us to see a wider dilemma for theory-heavy approaches: the *dilemma of demandingness.* It is plausible that possession of a healthy human brain with an intact global broadcast network suffices, given the actual laws of nature, for a capacity for conscious experience. The claim to sufficiency has substantial empirical support, rooted in the ability of adult humans to report their conscious experience of a stimulus. However, possession of a full human global broadcast network is a cognitively demanding sufficient condition that no non-human animal can meet, and the global workspace theory does not tell us how much we can weaken these demands and still have a sufficient condition. The result is that the theory cannot settle disputes about animal consciousness. Other theories, such as Merker’s, posit a much less demanding sufficient condition. They have more relevance to disputes about animal consciousness because the putative sufficient condition can be possessed by animals without a cortex, and is likely to be possessed by a wide range of animals. Yet these theories inevitably have much weaker evidential support in the human case, because they inevitably rely on evidence from subjects who cannot report their experiences.

The general dilemma is this: strong sufficient conditions for consciousness will not get us very far in making inferences about cases other than humans who can report their experiences, if they get us anywhere. Yet, as we formulate increasingly weaker conditions, the evidence from humans that they amount to a sufficient condition becomes weaker, and the positive case for animal consciousness becomes correspondingly weaker.

## 4 The Theory-Neutral Approach

This dilemma pushes us in the direction of a theory-neutral approach. What if we could resolve disputes about consciousness in non-human animals *without* first committing to a theory of consciousness in humans? Couldn’t we then use our conclusions about the distribution of consciousness to develop better theories? Tye, in *Tense Bees and Shell-Shocked Crabs* (2016a), has attempted such an approach, and this will be my focal example. I note, however, that theory- neutral approaches are popular in the literature on animal pain, where many authors have attempted to produce lists of markers of pain without assuming any underlying theory of consciousness (Bateson, 1991; Sneddon, Elwood, Adamo, & Leach, 2014; Varner, 2012). I do not think these authors succeed in avoiding substantial theoretical assumptions—in particular, I think they rely on assumptions about the function of conscious pain—but I will not argue for this here.

Tye is known for the “PANIC” theory of consciousness (Tye, 1995), a relative of the global workspace theory, but in his recent work he deliberately avoids any reliance on that theory. He employs what he calls “Newton’s rule”, or the “same effect, same cause” principle:

Suppose … that humans and nonhuman animals engage in the same or very similar behavior B… given the same noxious stimulus S. Why do humans produce behavior B, given S? Because *S* elicits the feeling of pain in them, and the feeling of pain causes B. …

Turning now to the case of nonhuman animals, I am entitled to infer that the feeling of pain causes behaviorB in them too unless I have a defeater to that inference, that is, a reason to believe that a causal story is operative in those animals that cannot be reconciled with the causal story that adverts to pain. (Tye, 2016a, p. 75, italics added)

This principle starts to unravel when we pull on the notion of a “defeater”. The defeaters clause is needed to avoid credulousness. If we say that, whenever we find in an animal any instance of a behaviour that in humans would have a conscious experience among its causes, we are *always* entitled to infer that the behaviour is also caused by a conscious experience in the animal, we would become credulous. For example, learned avoidance behaviour, in humans, often has a conscious experience among its causes: we avoid various noxious stimuli because they are associated with unpleasant experiences. But learned avoidance behaviours are found in the spinal cords of rats that have been disconnected from the brain (Allen, Grau, & Meagher, 2009), and in the nematode worm Caenorhabditis elegans, an animal 1mm in length with 302 neurons (Ha et al., 2010). I am not ruling out the possibility that disconnected spinal cords or nematodes are conscious. My claim is rather that it would be credulous to apply the “same effect, same cause” principle uncritically to these cases, and to infer without further ado that learned avoidance behaviours are caused by conscious mental states in these systems. To have a serious, empirically constrained debate, we need to allow, as Tye does, that considerations regarding cognitive and neural mechanisms may defeat the inference from surface behaviour to conscious experience.

Yet an approach of this type faces the question of what counts as a defeater.^8^ In one section, Tye asks: Is the absence of the cortex a defeater? Sceptics of consciousness in fish and invertebrates are inclined to say it is. Tye says it is not. To make this case, he relies on the same evidence Merker presents in favour of the midbrain theory: the evidence from carers of children with hydranen- cephaly. The implicit principle guiding Tye’s judgements about defeaters seems to be this: *to show that the absence of some neuroanatomical structure (such as the cortex) is not a defeater, we need to find evidence in humans showing that brains without that structure still have conscious experience.*

This principle nullifies the intended advantages of a theory-neutral approach. For if we can implement the above principle, then we have what we need to implement a theory-heavy approach. Clear evidence of consciousness in humans without a cortex would be compelling evidence for a brainstem-centred set of sufficient conditions, such as Merker’s. We motivated theory- neutrality by noting the paucity of evidence regarding consciousness without a cortex in humans, but now we see that a theory-neutral approach (at any rate, Tye’s version of it) does not escape reliance on that evidence. Critics who are concerned about the reliability of this evidence, and about Merker’s theory, will not find their concerns to be dispelled by Tye’s approach.

When Tye turns to arthropods (the “tense bees” and “shell-shocked crabs”) he rejects the suggestion that there are defeaters in this case (Tye, 2016a, pp. 150-155), even though a critic concerned by the dramatic neuroanatomical differences between vertebrates and arthropods could point to far more than the absence of a cortex. Strictly speaking, insects have no midbrain—only a complex that is functionally analogous to the vertebrate midbrain in some functional respects, as Barron and Klein emphasize. In the absence of a detailed background theory such as Merker’s (a theory that says: these are the respects that *matter* for consciousness), there is no principled reason for Tye to deny that radical differences of neuroanatomy throughout the entire brain can be defeaters.

What is the broader lesson here? It is the *inescapability of theory.* To avoid credulousness, theory- neutral approaches to animal consciousness need to get some grip on the defeaters: the minimum “system requirements” for consciousness or the negative markers of consciousness: the grounds, if any, on which we can exclude nematodes, plants, paramecia, amoebas and bacteria. But to get that kind of grip, the approach needs a background theory after all.^9^ In fact, the implicit background theory will need to be, in one sense, more ambitious than explicit theories such as the global workspace theory, because this theory will have to say something about minimum requirements and/or negative markers, not just sufficient conditions and positive markers.^10^ This is, I suggest, an even harder task. The defender of the supposedly theory-neutral approach will be unable to point to a solid, uncontested evidence-base for any such background theory, and the approach will fail to resolve disputes about contested cases.^11^

## 5 A Way Forward: The Theory-Light Approach

We have a dialectic with a whirlpool and a rock. The rock is the dilemma of demandingness, inevitably faced by theory-heavy approaches. The whirlpool is the inescapability of theory: it’s a whirlpool that spits you out in the direction of the rock. I will now propose an approach which synthesizes ideas from, among others, Allen (2004), Ginsburg and Jablonka (2019) and Andrews (2020), Shea and Bayne (2010), Shea and Frith (2016). The aim is to plot a middle course between the whirlpool and the rock by developing a *theory-light* approach to animal consciousness.^12^

A theory-light approach avoids committing to a fully specified theory of consciousness in humans, but it does not eschew theoretical commitments entirely. Instead, it commits to a broad hypothesis about the relation between phenomenal consciousness and cognition that is compatible with a wide range of more specific theories. The hypothesis I have in mind I call the *facilitation hypothesis.* The motivating idea is that phenomenal consciousness does *something* for cognition, given the actual laws of nature, but precisely what it does is a question to which we do not yet have definitive answers. More specifically, the facilitation hypothesis states that;

*Phenomenally conscious perception of a stimulus facilitates, relative to unconscious perception, a cluster of cognitive abilities in relation to that stimulus.*

The facilitation hypothesis involves a comparison between the processing that occurs when a stimulus is perceived consciously and the processing when it is perceived unconsciously. “Facilitates” should be read as “facilitates, holding all else fixed”. The claim is that, holding all else fixed (e.g. the stimulus, the difficulty of the task), a cluster of cognitive abilities is facilitated when the stimulus is perceived consciously. This is hypothesized to be a general truth about how conscious perception relates to cognition, given the actual laws of nature.

The term *cluster* is significant: the claim is not that there is a single positive marker of conscious experience (a “litmus test”), or a checklist of markers that are to be investigated separately. The claim is that there are multiple consciousness-linked cognitive abilities which cluster together, in the sense that there will be robust correlations between them: the abilities will come and go together, co-varying in a way that depends on whether or not a stimulus is consciously perceived. The strongest case for consciousness comes from finding a cluster of consciousness-linked cognitive abilities that robustly co-vary across multiple timescales. They will be strongly associated within a single experiment, during the development and ageing of a single individual, between organisms within a species, and across species.^13^ The existence of such a cluster is a theoretical commitment, but it is far from a completely specified theory of human consciousness (hence “theory-light”).

I contend that all currently popular theories of consciousness are compatible with the facilitation hypothesis. The facilitation hypothesis is compatible with theories that focus on the cortex and with theories that focus on the midbrain, since a midbrain-centric theory can still allow that consciousness facilitates cognitive abilities. It is compatible with theories that emphasize global broadcast and with theories that emphasize a mechanism causally upstream of global broadcast, such as fragile short-term memory (Block, 2007, 2011), because mechanisms upstream of global broadcast may still facilitate cognitive abilities (e.g. by providing informationally rich inputs to downstream processing). It is compatible with first-order representational theories (e.g. Dretske, 1995; Tye, 1995) and with higher-order theories (e.g. Carruthers, 2000; Rosenthal, 2005). Defenders of these theories are likely to disagree about the nature of the cognitive abilities that are facilitated by consciousness, and may therefore be led to make incompatible predictions about these abilities, but they can still agree that a relation of facilitation holds between consciousness and some cluster of cognitive abilities.

I also take the facilitation hypothesis to be compatible with the integrated information theory (IIT) (Tononi, Boly, Massimini, & Koch, 2016). This is not the place for detailed discussion of IIT. There is room for debate about how a distinction between conscious and unconscious perception can even be drawn within IIT given that, notoriously, the theory ascribes consciousness to very simple non-living systems such as photodiodes (Tononi & Koch, 2015, p. 11). However, there is room within the theory for a distinction between perceptual processing that occurs within the brain region of maximal integrated information (“maximal ø”) and perceptual processing that occurs outside that region. Koch, Massimini, Boly, & Tononi (2016) have proposed a “posterior cortical hot zone” as the region of maximal ø. What matters for our purposes is that it is compatible with IIT to claim that perceptual processing in the region of maximal ø facilitates, relative to perceptual processing outside that region, a cluster of cognitive abilities. For example, it may do so by providing informationally rich inputs to downstream processing.

Yet the facilitation hypothesis is not theory-neutral: some possibilities are ruled out from the beginning. One possibility that is ruled out is *cognitive epiphenomenalism* about conscious perception: the view that conscious (as opposed to unconscious) perception of a stimulus has no consequences whatsoever for cognition. On such a view, all cognitive abilities performable on consciously perceived stimuli could also be performed, at the same rate and with the same reliability, on unconsciously perceived stimuli. It is enough to say that the general objections to epiphenomenalism (Robinson, 2019) apply to this view. We can report consciously perceived stimuli but not unconsciously perceived stimuli, and it is a significant challenge to construct a theory of consciousness that explains why this is the case without positing a causal relationship between the experience and the report.

There is a view in the vicinity of cognitive epiphenomenalism on which conscious perception has only *one* cognitive manifestation: accurate verbal report. On such a view, conscious perception facilitates no cognitive ability *except* the accurate sharing of my perceptual states with others. This is still a valuable ability to have, since groups in which agents can share their perceptual states in this way can make better joint decisions (Frith & Metzinger, 2016, p. 214). A critic might argue that it is assuming too much to assume that a *cluster* of distinct cognitive abilities is facilitated by conscious perception.

But I maintain that the facilitation hypothesis can be empirically motivated. We already have some good, scientifically plausible candidates for abilities other than verbal report that are facilitated by consciousness. Here I will briefly review three such candidates.

One good candidate (as emphasized by Allen, 2004, 2013; Dehaene, 2014; Koch, 2004) is *trace conditioning.* Trace conditioning is a form of classical conditioning in which the two stimuli are separated by a temporal interval. For example, you might hear a tone in your ear and, one second later, feel a puff of air aimed at your eye. Clark and Squire’s (1998, 1999) work on trace eyeblink conditioning shows that, while humans can learn the association between the tone and the puff of air one second later, humans can only learn this association when *consciously aware of the stimuli and the temporal interval between them* (see also Clark, Manns, & Squire, 2001, 2002). If subjects are given a distracting task such that, when asked later, they show no reportable awareness of the relation between the stimuli, they can still do standard classical conditioning (where the associated stimuli are contemporaneous) and even delay conditioning (where the stimuli are not fully contemporaneous, but do overlap), but they cannot do trace conditioning. They cannot learn the temporal interval. This points towards conscious experience playing a role in facilitating temporal cognition and the learning of temporal relations.

One might ask: how reliable are these results? The central finding that human subjects cannot do full trace eyeblink conditioning when unaware of the stimulus contingencies was replicated by Knuttinen, Power, Preston, and Disterhoft (2001) and Bellebaum and Daum (2004), but Knuttinen et al. (2001) failed to replicate the finding that delay conditioning was *not* facilitated by awareness of stimulus contingencies. This remains a source of debate, with some critics maintaining that *all* human associative learning requires conscious awareness of stimulus contingencies (Lovibond & Shanks, 2002; Lovibond, Liu, Weidemann, & Mitchell, 2011; Mitchell, De Houwer, & Lovibond, 2009; Weidemann, Satkunarajah, & Lovibond, 2016). Yet even if delay conditioning also turns out to be facilitated by consciousness, this is no challenge to the idea that trace conditioning is facilitated by consciousness.

A second candidate ability is *rapid reversal learning.* Reversal learning involves learning a relationship between two stimuli, then learning the opposite relationship when it is reversed by the experimenter. A study by Travers, Frith, and Shea (2018), involving visual cues, suggested that “subjects were only able to adapt [rapidly, within up to 100 trials] to reversals of the cue-target contingencies … when consciously aware of the cues.” (Travers et al. 2018, p. 1698) Subjects were presented with arrow-heads (“ < < ” or “ > > ”) that were either backward masked or unmasked. They were subsequently shown a target and instructed to indicate which side of the screen the target was on. The arrowheads primed a response in the direction they pointed, regardless of whether they were masked or not. But subjects who consciously perceived the arrowheads were able, in addition, to learn the association in the current situation between the direction of the arrows and the location of the target. When the association was reversed, the response patterns of the conscious perceivers, but not the unconscious perceivers, changed. Because the subjects were tested in blocks of 100 trials each, this is testing for rapid learning without ruling out the possibility of unconscious learning over thousands of trials. A similar but more subtle ability involves rapidly learning to treat cues as less reliable when they become less reliable: for example, learning that a cue which used to predict a target with 90% accuracy is now only 70% accurate. Travers et al. found no evidence of this ability when the cues were unconsciously perceived. This points towards conscious experience facilitating fast associative learning in the face of novel, unpredictable changes in stimulus contingencies.

A third candidate ability is *cross-modal learning.* It is an idea incorporated by the global workspace theory, but not unique to that theory, that conscious experience facilitates the learning of associations that cut across sense modalities (Mudrik, Faivre, & Koch, 2014). For example, a consciously experienced odour can be associated with a consciously experienced sound, or with a consciously experienced visual stimulus, and so on. The facilitation claim amounts to the claim that cross-modal learning is at least *substantially easier,* as indicated by its speed and reliability, when the stimuli are consciously experienced than when they are unconsciously perceived. We need not claim that cross-modal learning is *impossible* when the stimuli are unconsciously perceived, only that it is harder. A hypothesis of this type is defended by Palmer and Ramsey (2012), who argue that “cross-modal effects can occur in the absence of consciousness, but the influencing modality must be consciously perceived for its information to cross modalities” (p. 353). They motivate this claim with an experiment based on the McGurk effect: when the visual stimulus (a person’s lips) was unconsciously perceived but blocked from conscious awareness by flash-suppression, the McGurk effect vanished.

Palmer and Ramsey’s thesis has recently been challenged by Scott, Samaha, Chrisley, & Dienes (2018), who present results suggesting that subjects *can* learn associations between auditory and visual stimuli even when both are perceived unconsciously. However, it is not clear that this experimental protocol successfully excluded those subjects who could hear the auditory stimulus consciously. The auditory stimulus was a spoken name, and Scott et al. excluded those subjects who reported hearing the name, but may not have excluded subjects who consciously perceived a *sound* (i.e. a phoneme with certain auditory properties) without being able to recognize that sound as a *name*.^14^ The evidential picture is, at present, inconclusive.

These three examples are enough to illustrate the “theory-light” strategy. Without committing to any particular theory of consciousness, we can investigate, in humans, the question of which cognitive abilities are facilitated by conscious perception. If we have only one such ability, and find that one ability in the target non-human species, a critic will say: that could be done without consciousness, even if it happens to involve consciousness in humans. So we need a *cluster* of correlated abilities, not just one, in order to build up a case that is harder for the critic to resist. The larger and more diverse the cluster, the stronger the case will be.

Once we have constructed, on the basis of evidence from humans, a tentative, defeasible hypothesis about the cluster of consciousness-linked abilities, the next step in the theory-light approach is to look for the cluster in the target species of nonhuman animal. Some elements of the cluster will, inevitably, be absent. We will not find verbal report. What we might find is a substantial fraction of the cluster. For example, we might find that bees can do trace conditioning of the right kind, reversal learning of the right kind, and cross-modal learning of the right kind. I say “of the right kind” in each case as an acknowledgement that more work is still needed here to pin down the precise type of each ability that is linked to consciousness in humans.

This will still not be enough to convince a reasonable critic, who will say: I’m afraid I can seriously envisage *all* of those abilities occurring without conscious experience, even though they are all facilitated by conscious experience in humans. You’ve shown the abilities are *present,* but you haven’t shown their *facilitation by consciousness.* This is a fair criticism, but we can overcome it. The next step in the theory-light approach should be to investigate protocols with the potential to cause unconscious perception in the animal: backward masking, the attentional blink, flashsuppression, distracting tasks, and so on. For brevity, I will refer to this whole family of procedures as “masking”. We need to find out whether the identified cluster of putatively consciousness-linked abilities is *selectively switched on and off* under masking in the same way it is in humans.

For example, in humans, presenting a tone subliminally appears to switch off trace conditioning while leaving delay conditioning in place. We can ask: is the same true of our target species of animal? Do we see a similar pattern of sensitivity to masking? When the stimulus is masked, does this selectively switch on and off the entire cluster of consciousness-linked abilities?

Our critic will wonder: *How could you ever show that a stimulus had been successfully masked, in the absence of verbal report?* But here too, we can respond. Suppose we find one ability (e.g. trace conditioning) and a putative masking protocol that selectively switches this ability off. We can then ask: does the same putative masking protocol also selectively switch off all the other abilities in the cluster (e.g. cross-modal learning and rapid reversal learning) while leaving less demanding cognitive abilities (such as delay conditioning and unimodal learning) unaffected? If so, this pattern of selective switching off, found across the whole cluster of abilities, simultaneously supports both the claim that the abilities are consciousness-involving and the claim that the putative masking protocol really was masking the stimulus. In short, there will be reciprocal evidential relations between the putative mask and the cluster of putatively consciousness-linked abilities.

If we gather all this evidence for our target species, we will have the evidential basis for a scientifically credible inference to the best explanation to the presence of consciousness in that species. We will have started with an empirically supported hypothesis about the cluster of cognitive abilities that is linked to consciousness in humans, found evidence of that cluster in the target species, and found the same pattern of sensitivity to masking.

All of this, so far, involves investigating a single target species. Further relevant evidence would come from casting our phylogenetic net more widely, and investigating whether the cluster of putatively consciousness-linked abilities is *strongly correlated across biological taxa.* Is the presence of one consciousness-linked ability strongly predictive of the presence of others? Do lineages which have evolved one of the key abilities (e.g. trace conditioning) very quickly and reliably evolve the others (e.g. cross-modal learning, rapid reversal learning)? In other words, do the abilities “switch on and off” together over evolutionary timescales, as well as on the timescale of the cognitive functioning of an individual animal? Assembling an evidence base for rigorous cross-species comparisons is a goal for the long term, but a feasible one.

Suppose we find evidence of a cluster of consciousness-linked abilities, switching off and on together with masking, and coming and going together over phylogenetic time. This would, I suggest, allow a much more secure inference to the best explanation to the presence of consciousness than any single marker in isolation, or even any checklist of markers that have not been shown to reliably cluster together. In creatures with nervous systems radically different from our own, in which neuroanatomical evidence is likely to be of limited use, this sort of cognition-based case is, I suspect, the strongest sort of evidence of consciousness we could obtain.

If we were to reach this position, our critic would have to fall back on the idea that a cognition- based case, no matter how carefully constructed, cannot be evidence of consciousness. They would have to cast doubt on what the facilitation hypothesis presupposes: that there is a close, albeit poorly understood, causal relationship between phenomenal consciousness and cognition. But it is not clear that animal consciousness research should be under any obligation to convince that kind of critic. Such a critic would primarily be objecting to the project of studying consciousness using cognitive science methods, whether in humans or in non-human animals, and their concern would not be specific to the animal case.

## 6 The Approaches Compared

I suggest that the theory-light approach provides the middle path we need between the mistake of assuming a controversial theory of consciousness at the outset and the mistake of insisting that behavioural evidence alone, gathered without any guiding theoretical commitment, will do the job. We should look at cognition, and do so with a specific focus on those abilities that we have reason to believe are facilitated by conscious perception in humans.

How does the theory-light approach avoid the pitfalls of theory-heavy and theory-neutral approaches? Let’s return to the dilemma of demandingness. When we try to formulate sufficient conditions for consciousness based on evidence from humans, we find there is a trade-off between the relevance of these conditions to animals and the strength of the evidence for their sufficiency. The theory-light approach avoids the dilemma by avoiding altogether the attempt to construct sufficient conditions for consciousness. At no point is it claimed that the cluster of cognitive abilities, or any part of the cluster, is nomologically sufficient for consciousness. Nor is there any significant role for neural correlates of consciousness (NCCs), since there is a presumption that these are likely to vary a great deal across biological taxa. Invertebrates lack even the basic forebrain-midbrain-hindbrain structure of the vertebrate brain. There are structures in insects analogous to the midbrain, as emphasized by Barron and Klein (2016), but these do not provide a convincing case for consciousness, for the reasons outlined in Section 3.^15^ The shift of emphasis away from NCCs is a significant point of contrast with the theory-heavy approach, in so far as the main scientific theories of consciousness (such as the global workspace theory and the midbrain theory) are all invested to at least some degree in the project of identifying NCCs.

The cognitive abilities are not intended as jointly sufficient conditions, but as markers. A cluster of symptoms does not suffice for a disease, but provides evidence of it, and the evidence is stronger when more of the cluster is found.^16^ This may be the best evidence we can have in the absence of a reliable blood test or genetic test. The relation between consciousness and the cluster of consciousness-linked cognitive abilities should be understood in the same way. The larger the fraction of the cluster we find in a given species, the stronger the case for consciousness will be in that species. In this respect, the theory-light approach tries to capture what is attractive about the theory-neutral approach.

There may be cases in which we find only a small part of the cluster (one or two capacities), and in these instances the case for consciousness will be weak. It’s a possible outcome of pursuing the theory-light approach that it will only ever deliver this sort of weak, inconclusive evidence in invertebrates, because no invertebrate species possesses a large enough fraction of the cluster to allow a strong case. That would be a disappointing result—but it is, inevitably, impossible to know in advance what the empirical outcome of a methodological strategy will be (Section 7 discusses the current state of the evidence for bees).

The problem with the theory-heavy approach is not that theories are undesirable, but that it commits prematurely to a specific theory at an early stage in inquiry, before we have evidence of the right type from across the animal kingdom. If the theory-light approach is pursued for a sustained period, the result may be a body of evidence that can be used to construct better theories. For example, we may ultimately be able to argue (by drawing on both cognitive and neurobiological evidence) that a core global broadcast mechanism (with some but not all of the features of the human global workspace) is present in all animals that possess the cluster of consciousness- linked abilities and explains why the abilities cluster together. This would lend some support to an ambitious version of the global workspace theory that emphasized the sufficiency of this core mechanism for consciousness, at least in living biological organisms.

The theory-light approach departs from the theory-neutral approach by accepting the inescapability of theory and making a minimal theoretical commitment. This commitment highlights a specific class of properties—cognitive abilities—as the place to look for outward manifestations of phenomenal consciousness. It also highlights a specific causal relation: facilitation. Because the approach does not regard all behavioural similarities as potentially relevant to the inference to consciousness, the theory-light approach can avoid credulousness without providing a theory of defeaters. Because the positive markers are carefully circumscribed by evidence from humans, there is no reason to think that the approach will ascribe consciousness too liberally in the absence of such a theory. In this respect, the theory-light approach tries to capture what is attractive about the theory-heavy approach.

## 7 Back to Bees

Let us return, then, to the case of bees. To what extent do we already have the evidence we need to implement the theory-light approach in this case, and to what extent is more evidence required? Let’s consider this in relation to the three candidates for consciousness-linked abilities presented in the previous section: trace conditioning, rapid reversal learning, and cross-modal learning.

There is some evidence, in insects, of a form of learning that has been described as “trace conditioning”. However, it differs in some respects from trace eyeblink conditioning. Szyszka et al. (2011) tested the ability of honey bees *(Apis* genus) to learn an association between an odour and a sugar reward presented around 5s later. The bees learned an association between the stimuli, in the sense that they anticipated food when presented with the odour. One might worry whether the odour was genuinely gone when the reward arrived: it is challenging to ensure a crisp stimulus offset when the stimulus is olfactory. Setting this aside, there is no evidence that the bees learned the *temporal interval* between the two stimuli, whereas Clark and Squire’s version of trace conditioning requires learning of a temporal interval: the eyeblink response must be timed to occur just long enough after the tone. We can distinguish here two kinds of trace conditioning: full trace conditioning, where a temporal interval between the stimuli is learned and the conditioned response is well-timed, and partial trace conditioning, where two stimuli separated in time are associated but the temporal interval between them is not learned and the conditioned response is not well-timed. Clark and Squire’s results show only the former ability to be linked to consciousness in humans.

There is separate evidence of interval timing in bumble bees *(Bombus* genus) from a study by Boisvert and Sherry (2006). Boisvert and Sherry built a chamber in which an initial sugar reward would be followed, after a specified time interval (of 6s, 12s, or 36s), by a second reward. In each case, the bees anticipated the reward, as shown by the bees’ waiting until (on average) a third of the interval had elapsed before attempting a proboscis extension response, waiting much longer when the interval was longer. As the time the reward was due came closer, the bees gradually increased the frequency of their proboscis extension responses. The bees learned temporal relations, in at least a coarse-grained way, but this is not trace conditioning because the initial stimulus was potent rather than neutral (it was a food reward). Even so, when we combine the evidence of partial trace conditioning in honey bees with Boisvert and Sherry’s evidence of interval timing in bumble bees, we have a case for regarding the hypothesis that bees can do full trace conditioning as a serious possibility worthy of investigation.

Rapid reversal learning, in contrast to full trace conditioning, is very well documented in bees. The ability of bees to learn a reversed association, and to do it in fewer than 100 trials, is not in doubt. For example, if one colour is associated with nectar and another is associated with its absence, and the colours are then reversed, bees will quickly learn the new colour-nectar contingencies (Strang & Sherry, 2014). There is, however, debate about whether the bees’ sensitivity to reversals improves or deteriorates over the course of multiple reversals. In rats, pigeons, goldfish (Shettleworth, 2010) and octopuses (Bublitz, Weinhold, Strobel, Dehnhardt, & Hanke, 2017), performance improves over successive reversals. This is often interpreted as a mark of intelligence: the animal is learning the need to change its behaviour at the first sign of a reversal. In honey bees, by contrast, performance seems to deteriorate (Couvillon & Bitterman, 1986; Mota & Giurfa, 2010). Yet there is evidence that, in bumble bees, it improves (Chittka, 1998; Sherry & Strang, 2014). Cognitive differences between honey bees and bumble bees remain poorly understood, and this appears to be a context in which they manifest in a surprising way (Sherry & Strang, 2015). Note, however, that, in Travers and colleagues’ experiment on humans, no rapid reversal learning of any kind was found when the visual stimulus was masked. Not just improvement over serial reversals but rapid reversal learning in its entirety was switched off by masking. If rapid reversal learning in bees could also be shown to be switched off by masking, this would be striking evidence.

Finally, is there evidence of cross-modal learning in bees? A study by Lawson, Chittka, Whitney, and Rands (2018) showed that bumblebees, having learned an association between a spatial scent pattern and a reward, showed a preference for *visual* arrays with the same pattern. Bees trained to associate a cross-shaped scent pattern with a reward subsequently preferred cross-shaped visual patterns, and bees trained to associate a circular scent pattern with reward subsequently preferred circle-shaped visual patterns. The bees seemed to be recognizing similarities between visual and olfactory patterns.^17^ This implies there is some association in the bee’s brain between how a circular scent pattern smells and how it looks, and how a square scent pattern smells and how it looks. This leaves open the question of how they recognize this similarity, and of the extent to which the cross-modal associations are formed by learning as opposed to being innate. Even so, it is a cross-modal effect. As with the McGurk effect, it would be informative to find out whether the effect could be abolished by a form of masking. Inferences from cross-modal effects to consciousness should be tentative, since, as noted above, we aren’t yet confident as to which kinds of cross-modal learning are facilitated by consciousness in humans. But evidence of cross-modal learning, especially if it is sensitive to masking, seems likely to form part of the eventual case for bee consciousness.

Although the evidence is building towards a strong future case for conscious experience in bees, we are still, it seems to me, some way off. As a result, I don’t side with either of the two camps in the current debate. The data that would be needed to settle disputes about whether bees are conscious or not do not yet exist. We have some relevant evidence, but we don’t, for example, have evidence regarding the sensitivity of the relevant cognitive abilities to masking, or evidence about the extent to which they correlate with each other across taxa.

Yet I see no reason to regard the debate as irresolvable, and I have set out a path that, if followed, could be used to advance it. What’s needed? At this stage, not more theory, and not more undirected data gathering. What is needed is a systematic search for consciousness-linked cognitive abilities, their relationships to each other, and their sensitivity to masking. Some evidence exists already, but most of these abilities need to be studied in more depth, and their sensitivity to masking remains unexplored. If we do ultimately find a substantial cluster of consciousness- linked abilities, showing the same pattern of sensitivity to masking as in humans, we would at that point have a strong cognition-based case that there is something it feels like to be a bee.

In our current state of uncertainty, a different question arises: how should we treat bees and other insects? In cases where some evidence of consciousness-linked cognitive abilities exists, no matter how tentative, there is a case for applying a version of the precautionary principle: we should err on the side of caution and take proportionate measures to safeguard the welfare of the animals concerned (Birch, 2017). But this is the beginning of a debate, not the end, because we have no real grip on what would constitute proportionate measures to protect the welfare of bees.

## Endnotes

1 I say “most of us” because some blind people navigate the world using sonar and may have some insight into what it’s like to echolocate, as Nagel (1974, p. 442, note 8) observes.

2 When I say an animal is “conscious” or “sentient” (and I will use the terms interchangeably in this article), I mean it has the capacity to form phenomenally conscious states. A phenomenally conscious state is a state there is something it feels like to be in. For discussion of attempts to define phenomenal consciousness more precisely than this, see Schwitzgebel (2016).

3 As we will see in Section 4, however, theory-neutral approaches tend not to live up to their theory-free billing.

4 A theory-heavy approach could proceed with a qualified version of this claim, such as the claim that, in general, entry to a global workspace *located in the brain of a living organism* confers phenomenal consciousness on mental representations. This would remain silent about consciousness in AI systems, but would have implications for non-human animals, infants, and patients in a minimally conscious state. But it would still run into the same problems as the ambitious interpretation.

5
Carruthers (2018a, 2018b, 2019) takes this type of worry much further, using it to motivate the radical claim that, if global workspace theory is a complete and fully reductive theory of human consciousness, then there is *no fact of the matter* as to whether non-human animals are conscious. There is simply gradual variation across the animal kingdom in the extent to which animals approximate the human global workspace, and that is all there is to say. I do not see a strong case for the conditional or for its antecedent, but this is a topic for another occasion (Birch, forthcoming).

6 A possible outcome of pursuing the “theory-light” strategy advocated in Section 5 is that, further down the line, we will be able to construct such an account (see Section 6). Shevlin (forthcoming) also considers how evidence from non-human animals may be used to augment the global workspace theory.

7 See Dehaene and Changeux (2011). I say “recent formulations” because Baars (1989) originally speculated that the midbrain might be an important part of the neural basis of the global workspace. However, Baars’ focus was mainly on the cognitive structure of the global workspace and not on its neural basis—an important difference of emphasis in relation to Dehaene and Changeux and in relation to Merker.

8 A similar concern has been raised by Klein (2017).

9 I have focussed here on one way in which the inescapability of theory manifests, but there are likely to be other ways. A theory-neutral approach not only needs some grip on defeaters—it also needs some grip on what makes two behaviours relevantly similar. These judgements of relevant similarity are likely to rely on a background theory of which functional/behavioural similarities are relevant and why (see also Klein, 2017).

10 In other respects, a theory of defeaters may be less ambitious than the global workspace theory. It need not commit to a detailed account of the cognitive or neural correlates of consciousness, but may instead draw on multiple more specific theories, attempting to tease out points of consensus about what counts as a defeater. The difficulty is that there are no points of consensus about what counts as a defeater. Some popular theories, such as the integrated information theory (Tononi et al., 2016), are willing to ascribe conscious experience extremely liberally to non-living entities.

11 For distinct but complementary criticisms of Tye’s approach, see Carruthers (2018b, pp. 182-186). Carruthers uses the failure of Tye’s theory-neutral approach to motivate a theory-heavy approach based on the global workspace theory. He finds that this too fails to settle questions about animal consciousness, or the reasons discussed in Section 2. This leads Carruthers to urge the wholesale abandonment of the attempt to settle these questions. But this is premature, as I argue in Section 5.

12 To elaborate briefly on these influences: Allen (2004) has emphasized the importance of consciousness-linked forms of learning, and in particular trace conditioning. Shea and Bayne (2010) and Andrews (2020) have emphasized the importance of looking for clusters of positive markers, not just a single marker (see also Shea, 2012). Shea and Frith (2016) have suggested that the relation between consciousness and cognition may be one of “facilitation”. Ginsburg and Jablonka (2019) have posited a close connection between learning and consciousness.

13 The importance of looking for clusters of markers, and not single markers or checklists, has been rightly emphasized by Shea and Bayne (2010) in the context of assessing patients in a minimally conscious or vegetative state (see also Bayne, 2018).

14 I thank Nick Shea for this point.

15 I therefore disagree with Klein and Barron when they write: “We don’t think any behavioural criteria on their own will ever differentiate animals that have subjective experience from those that don’t” (2016b, p. 7). I contend that cognitive abilities (manifested in behaviour) can provide evidence of subjective experience, once we have carefully delimited the relevant cognitive abilities using evidence from humans, and looked for correlations among the abilities and for sensitivity to masking. They can provide evidence even if we know little about the underlying neural mechanisms. However, understanding the mechanisms is likely to help us build better theories.

16
Shea and Bayne (2010) make a similar analogy. There is a close affinity between the theory-light approach and Shea and Bayne’s “natural kind approach” to human consciousness, noted earlier as an influence. However, Shea and Bayne (2010) try to do without even the minimal theoretical commitment embodied in the facilitation hypothesis, committing only to the claim that consciousness is a natural kind.

17 A study by Solvi, Guttierez Al-Khudairy, and Chittka (2020) found associations between tactile and visual patterns. Harrap, Lawson, Whitney, and Rands (2019) looked for similar transfer between temperature detection and vision, but did not find it.
